# Inhibitory mechanism of vortioxetine on CYP450 enzymes in human and rat liver microsomes

**DOI:** 10.3389/fphar.2023.1199548

**Published:** 2023-09-18

**Authors:** Yunyun Zhan, Anzhou Wang, Yige Yu, Jie Chen, Xinhao Xu, Jingjing Nie, Jingjing Lin

**Affiliations:** ^1^ Department of Pharmacy, The Affiliated Lihuili Hospital, Ningbo University, Ningbo, Zhejiang, China; ^2^ Department of Pharmacy, The Third Affiliated Hospital of Wenzhou Medical University, Wenzhou, Zhejiang, China; ^3^ Department of Pharmacy, The First Affiliated Hospital of Wenzhou Medical University, Wenzhou, Zhejiang, China

**Keywords:** vortioxetine, inhibition mechanism, drug-drug interaction, CYP450, UPLC-MS/MS

## Abstract

Vortioxetine is a novel anti-major depression disorder drug with a high safety profile compared with other similar drugs. However, little research has been done on drug-drug interactions (DDI) about vortioxetine. In this paper, the inhibitory effect of vortioxetine on cytochrome P450 (CYP450) and the type of inhibitory mechanism were investigated in human and rat liver microsomes. We set up an *in vitro* incubation system of 200 μL to measure the metabolism of probe substrates at the present of vortioxetine at 37°C. The concentrations of the metabolites of probe substrates were all measured by ultra-performance liquid chromatography tandem mass spectrometry (UPLC-MS/MS) method. It was found no time-dependent inhibition (TDI) of vortioxetine through determination of half-maximal inhibitory concentration (IC_50_) shift values. The enzymes and metabolites involved in this experiment in human and rats were as follows: CYP3A4/CYP3A (midazolam); CYP2B6/CYP2B (bupropion); CYP2D6/CYP2D (dextromethorphan); CYP2C8/CYP2C-1 (amodiaquine); CYP2C9/CYP2C-2 (losartan); and CYP2C19/CYP2C-3 (mephenytoin). We found that vortioxetine competitively inhibited CYP2C19 and CYP2D6 in human liver microsomes (HLMs) with inhibition constant (K_i_) values of 2.17 μM and 9.37 μM, respectively. It was noncompetitive inhibition for CYP3A4 and CYP2C8, and its K_i_ values were 7.26 μM and 6.96 μM, respectively. For CYP2B6 and CYP2C9, vortioxetine exhibited the mixed inhibition with K_i_ values were 8.55 μM and 4.17 μM, respectively. In RLMs, the type of vortioxetine inhibition was uncompetitive for CYP3A and CYP2D (K_i_ = 4.41 and 100.9 μM). The inhibition type was competitive inhibition, including CYP2B and CYP2C-2 (K_i_ = 2.87 and 0.12 μM). The inhibition types of CYP2C-1 and CYP2C-3 (K_i_ = 39.91 and 4.23 μM) were mixed inhibition and noncompetitive inhibition, respectively. The study of the above mechanism will provide guidance for the safe clinical use of vortioxetine so that the occurrence of DDI can be avoided.

## 1 Introduction

With the rapid development of the times and fast-paced life, the number of people suffering from depression is increasing year by year (Lam et al., 2014). The cognitive function of the brain will be greatly damaged after suffering from major depressive disorder, which will greatly affect people’s daily life and work (Pehrson and Sanchez, 2014). Vortioxetine, as a novel anti-major depressive disorder drug, may have two mechanisms at present: one is to inhibit serotonin transporter, and the other is to modulate the effects of several serotonin (5-HT) receptors (Pehrson and Sanchez, 2014). Based on the above mechanism of action, vortioxetine has certain therapeutic effects on cognitive impairment repair, sleep disturbance and chronic neuropathic pain (Frampton, 2016; Adamo et al., 2021). Clinical data show that vortioxetine does not need to adjust the therapeutic dose for these influencing factors such as gender and age, which means that the drug has greater clinical safety (Chen et al., 2018).

In addition to paying attention to the pharmacodynamics of the drug, we should also pay attention to its pharmacokinetics, such as what happens to vortioxetine in the body’s metabolism. Drug’s metabolism through the liver, gastrointestinal tract, lungs, skin, kidneys, etc., of which 90% of the drugs are metabolized by the liver (Arora et al., 2015). The reason is that there are a lot of cytochrome P450 (CYP450) enzymes in the liver, and these enzyme proteins are mainly used for the oxidation reaction of substances in the human body. In human, vortioxetine has been shown to be involved in the metabolism of CYP450 enzymes, including CYP2A6, CYP3A4/5, and CYP2C9, CYP2C19, and CYP2D6 (Hvenegaard et al., 2012). Its main metabolic enzyme was CYP2D6, followed by others (Hvenegaard et al., 2012). According to the current statistics, there is no significant difference about using vortioxetine between Chinese people and non-Chinese people whether they were strong metabolizers or poor metabolizers (Miao et al., 2019). But whether drug-drug interactions (DDI) affect its metabolism is now poorly understood.

Here we explored the type of mechanism of inhibition by vortioxetine on CYP3A4/3A, CYP2B6/2B, CYP2C8/2C-1, CYP2C9/2C-2, CYP2C19/2C-3, and CYP2D6/2D in human liver microsomes (HLMs) and rat liver microsomes (RLMs), in which the substrates corresponding to each enzyme were midazolam, bupropion, amodiaquine, losartan, mephenytoin, and detromethorphan. We used an ultra-performance liquid chromatography tandem mass spectrometry (UPLC-MS/MS) as the analytical instrument to assess the specific mechanism of inhibition of vortioxetine on CYP450 enzymes. However, no related results have been reported before.

## 2 Materials and methods

### 2.1 Materials and regents

HLMs and RLMs all were purchased from iPhase Pharmaceutical Services Co., Ltd. (Beijing, China). Metabolic probe substrates and their metabolites including midazolam, 1-hydroxymidazolam, bupropion, hydroxy bupropion, amodiaquine, N-desethyl amodiaquine, losartan, E-3174, mephenytoin, 4-hydroxy mephenytoin, dextromethorphan, and dextrorphan were purchased from Shanghai Canspec Scientific Instruments Co., Ltd. (Shanghai, China). The internal standard diazepam and vortioxetine were purchased from Beijing Sunflower Technology Development Co., Ltd. (Beijing, China). The reduced nicotinamide adenine dinucleotide phosphate (NADPH) used in the incubation system was purchased from Roche Pharmaceutical Ltd. (Basel, Switzerland). Methanol and acetonitrile used for UPLC-MS/MS were purchased from Merck (Darmstadt, Germany). Ultrapure water was obtained from Millipore (Bedford, MA, United States) by using a Milli-Q A10 purification system.

### 2.2 Microsomal incubation procedure

To study the inhibitory effect of vortioxetine on specific enzymes, *in vitro* incubation system of 200 μL was established, and the incubation temperature was 37°C. The components of the incubation system were as follows: 0.20 mg/mL HLMs or 0.20 mg/mL RLMs, 0.1 M Tris-HCl, vortioxetine, and the probe substrates of each enzyme. In the study of K_m_’s (Michaelis–Menten constant) experiment, midazolam (1, 2, 5, 10, 20, 50 and 100 μM in HLMs and RLMs) corresponded to CYP3A4/3A; bupropion (10, 20, 50, 100, 200, 500, 1,000 and 2000 μM in HLMs; 1, 2, 5, 10, 20, 50, 100 and 200 μM in RLMs) corresponded to CYP2B6/2B; amodiaquine (5, 10, 20, 50, 100 and 200 μM in HLMs; 10, 20, 50, 100, 200, 500 μM in RLMs) corresponded to CYP2C8/CYP2C-1; losartan (1, 2, 5, 10, 20, 50, 100, 200 μM in HLMs; 10, 20, 50, 100, 200, 500 and 1,000 μM in RLMs) corresponded to CYP2C9/CYP2C-2; mephenytoin (20, 50, 100, 200, 500 and 1,000 μM in HLMs; 5, 10, 20, 50, 100, 200, 500 and 1,000 μM in RLMs) corresponded to CYP2C19/CYP2C-3; and dextromethorphan (1, 2, 5, 10, 20, 50 and 100 μM in HLMs and RLMs) corresponded to CYP2D6/CYP2D. After 5.0 min of preincubation in the shaking water bath, the reaction was started with the addition of 1.0 mM NADPH. The incubated temperature at 37°C for 30 min. After the incubation, the enzyme reaction was stopped by cooling at −80°C, and 200 μL of acetonitrile and 20 μL of internal standard diazepam (200 ng/mL) were added for protein precipitation. The solution was fully vortexed and centrifuged at 13,000 × rpm for 10 min, and the supernatant was collected for data analysis by UPLC-MS/MS.

The significance of half-maximal inhibitory concentration (IC_50_) was the concentration of the inhibitor to cause 50% inhibition of original enzyme activity. To explore the IC_50_, the concentrations of the inhibitor vortioxetine were all 0.01, 0.1, 1, 10, 25 and 50 μM, while individual substrate was set according to its corresponding K_m_ value.

### 2.3 IC_50_-shift experiments of vortioxetine

IC_50_-shift was used to investigate whether the drug produced time-dependent inhibition (TDI) of the enzyme. This inhibitory property belongs to the category of irreversible inhibition among the types of enzyme inhibition. The IC_50_-shift experiment was performed by preincubating vortioxetine with HLMs or RLMs for 30 min at 37°C in the absence and presence of 1.0 mM NADPH. The above mixture was then incubated for another 30 min. During the experiment, the individual substrates midazolam (4 μM), bupropion (40 μM), amodiaquine (3 μM), losartan (8 μM), mephenytoin (90 μM), and dextromethorphan (10 μM) were determined with vortioxetine IC_50_-shift in HLMs. In RLMs, the substrates midazolam (3.5 μM), bupropion (20 μM), amodiaquine (20 μM), losartan (15 μM), mephenytoin (400 μM), and dextromethorphan (20 μM) were determined with vortioxetine IC_50_-shift, which based on its K_m_ value. Other experimental conditions were the same as above.

### 2.4 Reversible inhibition of vortioxetine on CYP450

The inhibitory effect of the drug on different enzymes was different. The inhibitory mechanism of the inhibitor on the enzyme was generally used by different concentrations of the inhibitor against different concentrations of the probe substrate. The concentrations of the inhibitor and the concentrations of probe substrates in the experiment were estimated based on IC_50_ and K_m_ values, respectively.

In HLMs, the concentrations of the substrates midazolam, bupropion, amodiaquine, losartan, mephenytoin, and dextromethorphan were 1, 2, 4, 8 μM; 10, 20, 40, 80 μM; 0.8, 1.5, 3, 6 μM; 2, 4, 8, 16 μM; 20, 45, 90, 180 μM; 2.5, 5, 10, 20 μM, respectively. Corresponding to the concentration of vortioxetine were 2, 4, 9, 18 μM; 2, 4, 8, 16 μM; 4, 8, 15, 30 μM; 5, 10, 25, 50 μM; 0.6, 1.2, 2.5, 5 μM; 4.5, 9, 18, 36 μM, respectively.

In RLMs, the concentrations of the substrates midazolam and dextromethorphan were 0.8, 1.6, 3.5, 7 μM and 5, 10, 20, 40 μM, respectively, vortioxetine concentrations were 4, 8, 16, 32 μM. The concentrations of mephenytoin and amodiaquine were 100, 200, 400, 800 μM and 5, 10, 20, 40 μM, respectively, vortioxetine concentrations were 5, 10, 20, 40 μM. The losartan concentrations were 4, 8, 15, 30 μM, corresponding to vortioxetine concentrations of 0.08, 0.15, 0.3, 0.6 μM. Bupropion concentrations were 5, 10, 20, 40 μM, corresponding to vortioxetine concentrations of 2, 4, 8, 16 μM. The experimental conditions were the same as above.

### 2.5 UPLC-MS/MS conditions

All experiments results were detected by UPLC-MS/MS, which equipped with a Waters Acquity UPLC system (Milford, MA, United States) and a Waters Xevo TQ-S triple quadrupole tandem mass spectrometer with an electrospray ionization source (Milford, MA, United States). The chromatographic separation was performed on a Waters ACQUITY UPLC BEH C18 column (2.1 × 50 mm, 1.7 μm, Waters Corp.) at 40°C. The mobile phase was consisted of acetonitrile (A) and 0.1% formic acid in water (B). The gradient elution at a flow rate of 0.4 mL/min was conducted as follows: 0–0.5 min, 10% A; 0.5–1.0 min, 10%–90% A; 1.0–2.0 min, 90% A; 2.0–2.1 min, 90%–10% A, and 2.1–3.0 min, 10% A. The relevant analysis parameters were shown in Table 1.

**TABLE 1:** Analytical parameters for the metabolites and internal standard (IS).

Analytes	Ionization mode	Parent (*m/z*)	Daughter (*m/z*)	Cone (V)	Collision (V)
Hydroxy bupropion	ESI ^+^	256.0	238.0	20	10
4-hydroxy mephenytoin	ESI ^+^	235.1	150.0	15	20
Dextrorphan	ESI ^+^	258.0	157.0	55	40
E-3174	ESI ^+^	437.2	235.0	20	15
N-desethyl amodiaquine	ESI ^+^	328.0	282.8	20	15
1-Hydroxymidazolam	ESI ^+^	341.9	324.0	50	20
Diazepam (IS)	ESI ^+^	285.0	154.0	10	30

### 2.6 Statistical analysis

IC_50_ values and enzyme kinetic parameters were calculated by Graphpad Prism 6.0 (GraphPad software Inc., CA, United States). Lineweaver-Burk Plot was used to determine inhibitor patterns. Dixon plots of K_i_ values were obtained from the slope of the nonlinear regression line in the Lineweaver-Burk plot as a function of different inhibitor concentrations, while secondary plots for αK_i_ were obtained from the y-intercept of the nonlinear regression line in Lineweaver-Burk Plot versus concentration of different inhibitors. Data for each experiment were expressed as mean ± SD (n = 3).

## 3 Results

### 3.1 IC_50_-shift assay in HLMs and RLMs

According to the results in Table 2, vortioxetine was a potential inhibitor of CYP450. It was found that vortioxetine had medium inhibitory efficiency on CYP3A4, CYP2B6, and CYP2C19 in HLMs. In RLMs, vortioxetine had medium inhibitory efficiency on CYP2B, and strong inhibitory effect on CYP2C-2.

**TABLE 2:** The IC_50_ values on CYP450 in (−) NADPH or (+) NADPH with IC_50_ fold shift and inhibitory effects of vortioxetine on CYP450 in HLMs and RLMs.

	CYP isoform	IC_50_	IC_50_ values (μM)		IC_50_ fold shift	Inhibition type			
			(−) NADPH	(+) NADPH			Ki (μM)	αKi (μM)	α
HLMs	CYP3A4	9.31	15.52	13.65	1.14	Noncompetitive	7.26	6.82	0.94
	CYP2B6	8.94	5.38	6.69	0.80	Mixed	8.55	23.04	2.70
	CYP2C8	15.00	18.21	18.70	0.97	Noncompetitive	6.96	13.48	1.94
	CYP2C9	23.03	18.57	16.66	1.11	Mixed	4.17	66.5	15.94
	CYP2C19	2.40	5.88	2.83	2.08	Competitive	2.17	-	-
	CYP2D6	17.93	19.37	18.35	1.06	Competitive	9.37	-	-
RLMs	CYP3A	16.37	20.94	16.41	1.28	Uncompetitive	4.41	-	-
	CYP2B	8.89	10.11	5.30	1.91	Competitive	2.87	-	-
	CYP2C-1	22.17	19.78	9.97	1.98	Mixed	39.91	56.73	1.42
	CYP2C-2	0.37	8.97	37.47	0.24	Competitive	0.12	-	-
	CYP2C-3	20.28	24.63	5.21	4.72	Noncompetitive	4.23	12.54	2.97
	CYP2D	15.67	3.35	7.38	0.45	Uncompetitive	100.9	-	-

Note: IC_50_ fold shift value is the ratio of the IC_50_ value of (−) NADPH, to the IC_50_ value of (+) NADPH.

**FIGURE 1:**
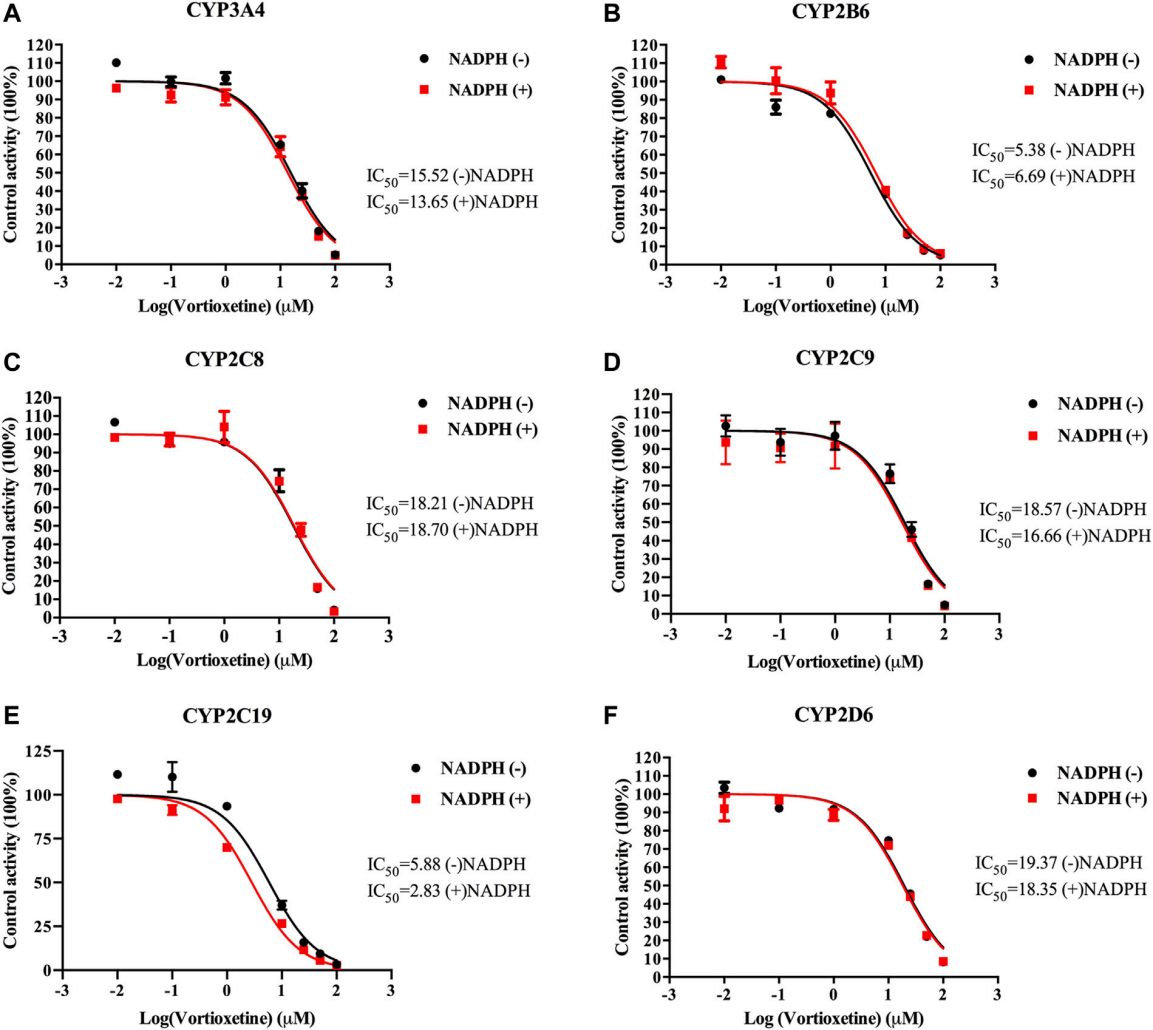
The IC_50_ shift curves of vortioxetine on the **(A)** CYP3A4, **(B)** CYP2B6, **(C)** CYP2C8, **(D)** CYP2C9, **(E)** CYP2C19 and **(F)** CYP2D6 activities in HLMs. The concentrations of vortioxetine were 0.01–100 μM. Values are expressed as mean ± SD of three samples.

**FIGURE 2:**
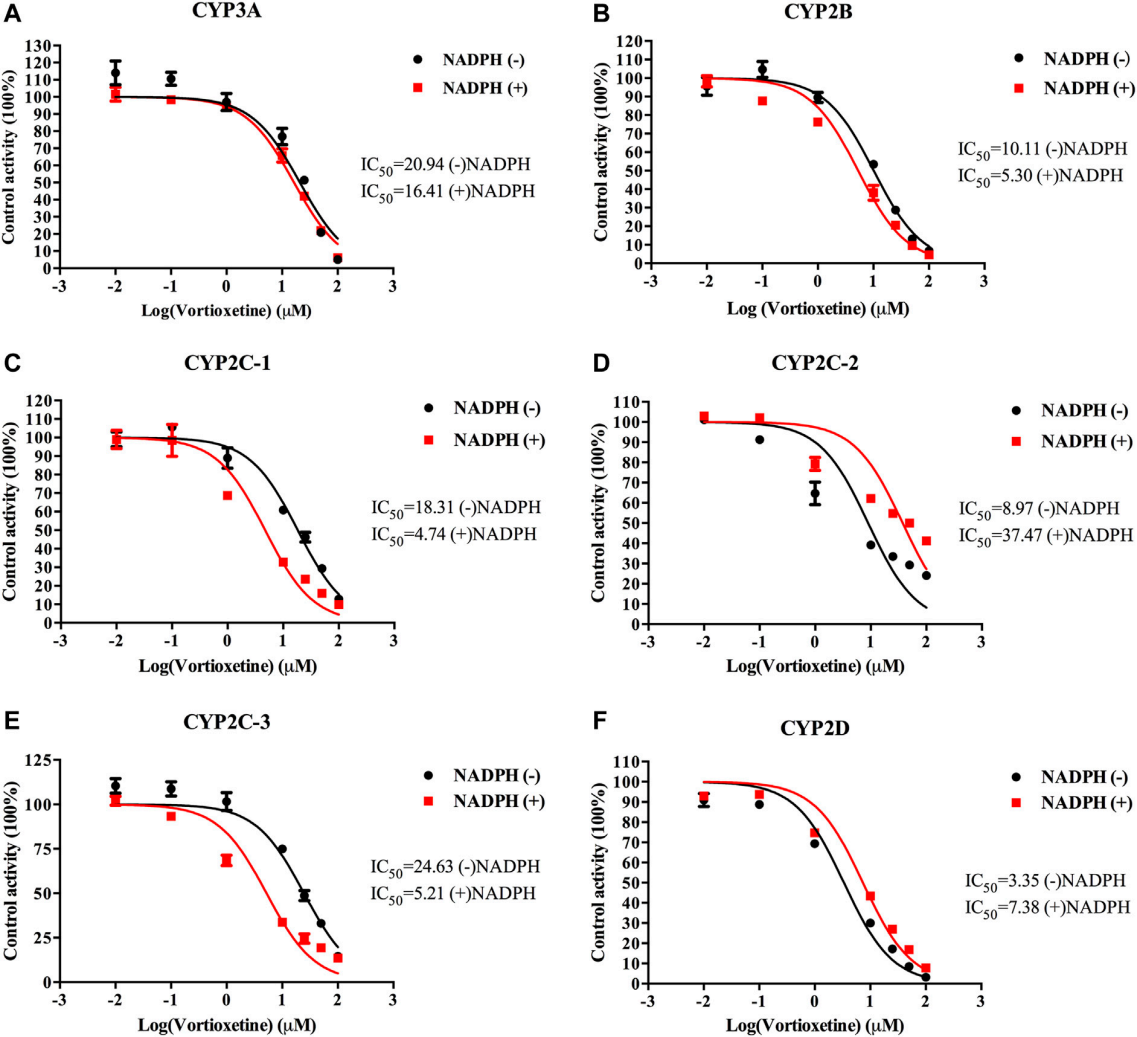
The IC_50_ shift curves of vortioxetine on the **(A)** CYP3A, **(B)** CYP2B, **(C)** CYP2C-1, **(D)** CYP2C-2, **(E)** CYP2C-3 and **(F)** CYP2D activities in RLMs. The concentrations of vortioxetine were 0.01–100 μM. Values are expressed as mean ± SD of three samples.

The value of IC_50_-shift was used to assess whether there was a TDI in the process of enzyme inhibition. As previously reported, when IC_50_-shift >10, there will be obvious TDI. According to the results, we found no TDI for CYP450 by vortioxetine (Table 2; Figures 1, 2). Particularly, the activity of CYP2C19 was significantly reduced with the NADPH in HLMs. Conversely, the activity of CYP2C-2 was significantly increased with the NADPH in RLMs.

### 3.2 Enzyme kinetic analysis of vortioxetine

**FIGURE 3:**
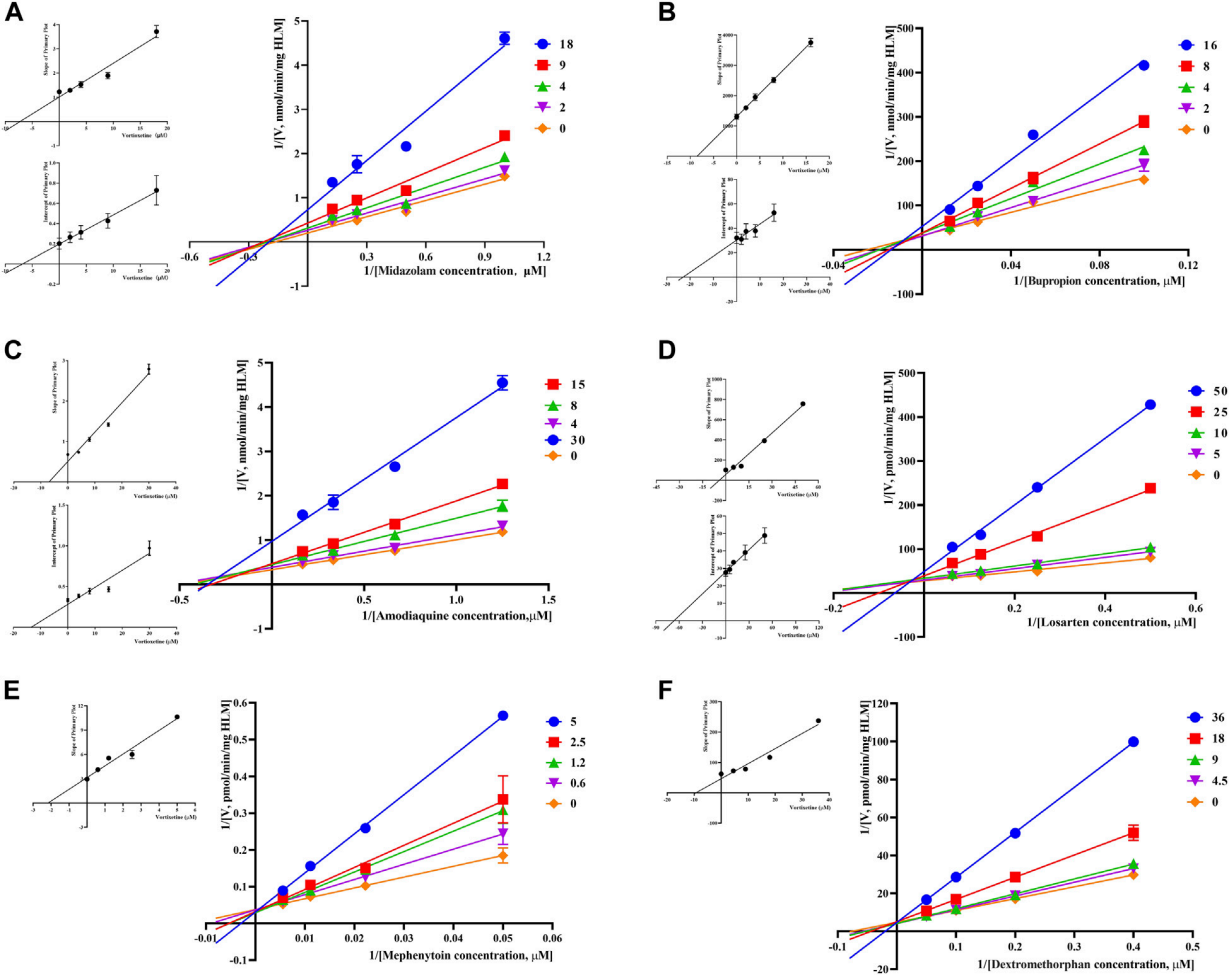
Primary Lineweaver–Burk plot, the secondary plot for K_i_ and the secondary plot for αK_i_ in the inhibition of CYP-mediated probe metabolism by various concentrations of vortioxetine in HLMs. **(A)** Midazolam was used at concentration of 2, 4, 9 and 18 μM; **(B)** bupropion was used at concentration of 2, 4, 8 and 16 μM; **(C)** amodiaquine was used at concentration of 4, 8, 15 and 30 μM; **(D)** losartan was used at concentration of 5, 10, 25 and 50 μM; **(E)** mephenytoin was used at concentrations of 0.6, 1.2, 2.5 and 5 μM; **(F)** dextromethorphan was used at concentrations of 4.5, 9, 18 and 36 μM. Each data point represents the mean of three samples.

**FIGURE 4:**
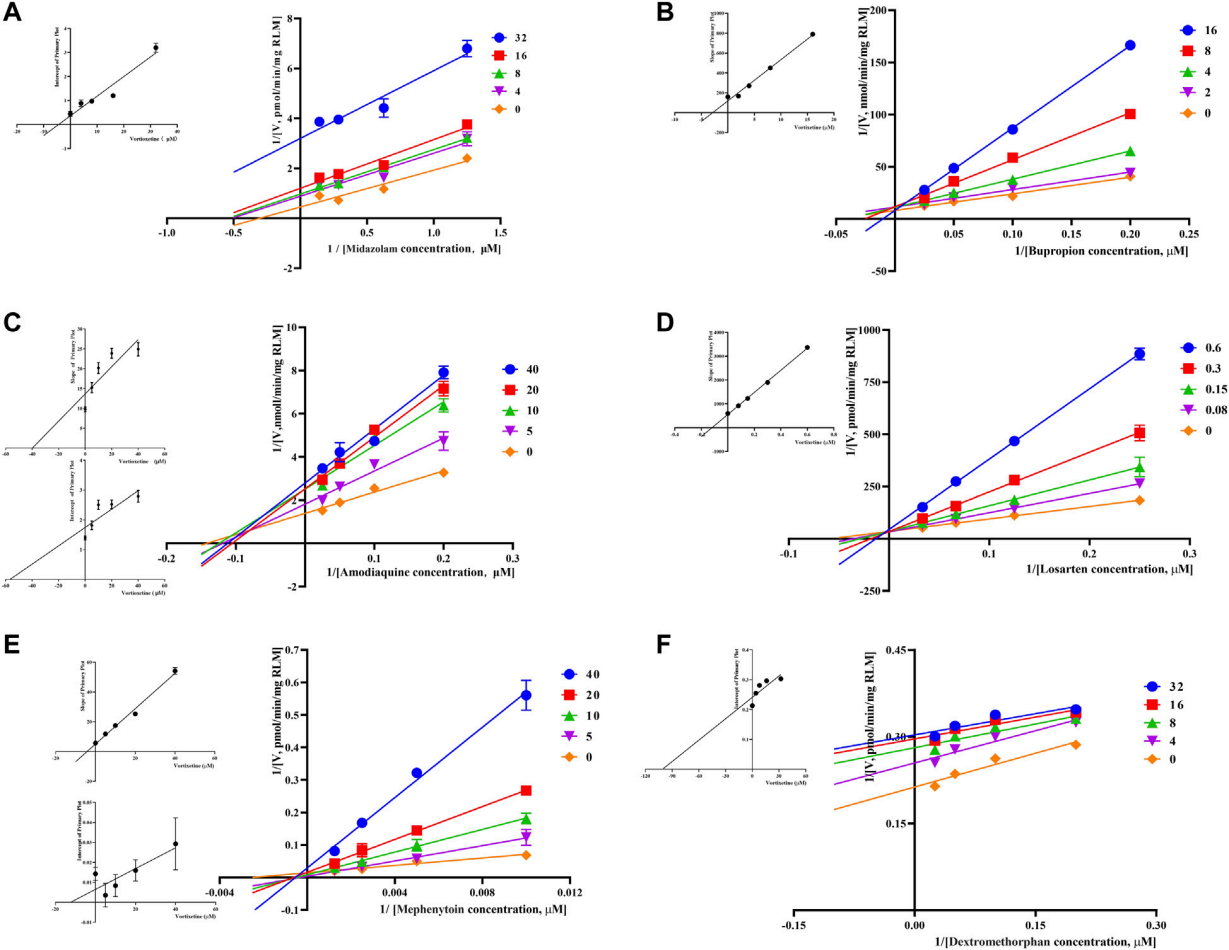
Primary Lineweaver–Burk plot, the secondary plot for Ki and the secondary plot for αKi in the inhibition of CYP-mediated probe metabolism by various concentrations of vortioxetine in RLMs. **(A)** Midazolam and dextromethorphan were both used at concentration of 4, 8, 16 and 32 μM; **(B)** bupropion was used at concentration of 2, 4, 8 and 16 μM; **(C)** amodiaquine and mephenytoin were both used at concentration of 5, 10, 20 and 40 μM; **(D)** losartan was used at concentration of 0.08, 0.15, 0.3 and 0.6 μM; **(E)** Mephenytoin was used at concentration of 5, 10, 20 and 40 μM; **(F)** Dextromethorphan was used at concentration of 4, 8, 16 and 32 μM. Each data point represents the mean of three samples.

Through the value of IC_50_-shift, we can preliminarily determine whether there was a TDI of vortioxetine on CYP450 enzymes. To further investigate the inhibitory mechanism of vortioxetine on the CYP450 enzymes, we used a series of concentrations of vortioxetine to react with the probe substrates and determine their metabolisms in HLMs and RLMs. In HLMs (Figure 3), the type of inhibition mechanism of vortioxetine on CYP3A4 and CYP2C8 was noncompetitive, with K_i_ values of 7.26 μM and 2.96 μM, respectively. The type of inhibition mechanism of vortioxetine on CYP2B6 and CYP2C9 was mixed, with K_i_ values of 8.55 μM and 4.17 μM, respectively. The inhibition mechanism of CYP2C19 and CYP2D6 was competitive, with K_i_ values of 2.17 μM and 9.37 μM, respectively. In RLMs (Figure 4), the type of inhibitory mechanism of vortioxetine on CYP3A and CYP2D was uncompetitive, with K_i_ values of 4.41 μM and 100.9 μM, respectively. The type of inhibitory mechanism of vortioxetine on CYP2B and CYP2C-2 was competitive, with K_i_ values of 2.87 μM and 0.12 μM, respectively. The inhibition mechanism type was mixed to CYP2C-1 with the K_i_ value of 39.91 μM and the inhibition mechanism type was noncompetitive to CYP2C-3 with the K_i_ value of 4.23 μM.

## 4 Discussion

Although current clinical data suggested that vortioxetine was a higher safety profile compared with other anti-major depressive disorder drug, polypharmacy cannot be avoided in most patients (Yee et al., 2018). DDI still cannot be ignored. Most of DDI occurred because a drug affected the activity of liver enzymes, resulting in toxic reactions caused by changed in the rate of metabolism of other drugs (Fowler and Zhang, 2008). Though we found that propafenone showed the inhibitory effect on vortioxetine metabolism in previous study (Xu et al., 2021), the mechanism of action of vortioxetine on liver drug enzymes has never been reported before. Here we firstly explored the mechanism of vortioxetine’s inhibition on CYP450 enzymes in HLMs and RLMs.

Judging the inhibitory strength of a drug on an enzyme was usually judged by the value of the IC_50_. According to the general standard, IC_50_ < 1 μM suggested strong inhibitory effect, 1 μM < IC_50_ < 10 μM suggested medium inhibitory effect, and IC_50_ > 10 μM suggested weak inhibitory effect (Jin et al., 2015). The results (Table 2) showed that vortioxetine was medium inhibitory effect on CYP3A4, CYP2B6 and CYP2C19 in HLMs, and medium inhibitory effect on CYP2B and strong inhibitory effect on CYP2C-2 in RLMs. As for other CYP450 enzymes, there were also weak inhibitory effects. Therefore, vortioxetine can be determined as a potential liver drug enzyme inhibitor. The mechanism of drug inhibition on CYP450 enzymes can be divided into reversible inhibition and irreversible inhibition (Lou et al., 2019). Reversible mechanisms are divided into competitive inhibition, noncompetitive inhibition, uncompetitive inhibition and mixed inhibition.

All the competition mode calculations in this experiment were obtained by Lineweaver-Burk. TDI in irreversible inhibition is the most commonly explored mode of inhibition today. Nowadays, there is no accurate conclusion about whether drugs have TDI in CYP450 enzymes, and the IC_50_-shift value is often used to determine whether there is TDI (Bao et al., 2018). It has been reported in the literature that TDI is a positive reaction when IC_50_-shift >10 (Grimm et al., 2009). The result of our experiment was that there was no significant change in IC_50_-shift detected after 30 min pre-incubation with NADPH or not. It can be concluded that vortioxetine has no TDI on the above six CYP450 isoforms.

Based on the above judgments, the inhibitory effects of vortioxetine on CYP450 enzymes studied in this paper were all reversible inhibition. We found that CYP2C19 and CYP2D6 were competitive inhibition, CYP3A4 and CYP2C8 were noncompetitive inhibition, and CYP2B6 and CYP2C9 were mixed inhibition in HLMs. Based on the above-mentioned inhibitory mechanism, it is of great help for the guidance of multiple drugs in clinical practice. Thus, in the clinic, the combination of vortioxetine and CYP450 substrate drugs should be avoided or monitored, even when these drugs are administered in therapeutic doses. For example, common gastric ulcer patients used proton pump inhibitors such as omeprazole or H_2_ receptor inhibitor cimetidine, which are inhibitors of CYP2C19 and CYP2C9, respectively (Miner et al., 2003; Burt et al., 2016). When these drugs were taken concurrently with vortioxetine, the patient’s blood concentration should be monitored or the dosage should be adjusted appropriately to avoid adverse reactions. The differences in IC_50_ value and inhibitory mechanism between HLMs and RLMs may also be caused by differences between species (Shimada et al., 1997; Komura and Iwaki, 2008; Nishimuta et al., 2013).

More studies are needed to interpret the role of vortioxetine in altering the response of drug-metabolizing CYP450 enzymes. The structural information may yield insights into the loss of activities of CYP450 enzymes and the differences in the inhibitory mechanisms, which provide further aids in understanding how vortioxetine influences protein function in biologic systems (Parikh et al., 2020). Previously, Pallan et al. team had obtained a crystal structure of human P450 21A2 in complex with progesterone, a substrate in adrenal 21-hydroxylation, and the structure of the human P450 21A2-substrate complex provides direct insight into mechanistic effects of genetic variants (Pallan et al., 2015b). Similar paper could also be found that several minor structural differences of x-ray crystal structures of zebrafish P450s 17A1 and 17A2, as well as with human P450 17A1, may be critical in understanding the basis of lyase function (Pallan et al., 2015a). Moreover, serotonin levels are controlled beside reuptake by the enzyme monoamine oxidase A (MAO-A) and that impaired MAO A leads to pathology that cannot be treated pharmacologically (Prah et al., 2020; Prah et al., 2022).

Vortioxetine at a single oral dose of 10 mg was clinically used in 14 healthy subjects with a C_max_ of 4.60 ng/mL and an AUC_(0-t)_ of 254.72 ngh/mL (Chen et al., 2018). Another study found that patients with severe liver impairment taking a single oral dose of 10 mg of vortioxetine, AUC_(0-t)_ was 10% higher and C_max_ 24% lower than healthy subjects, but it is not considered to adjust the clinical dose (2022). However, the plasma protein of vortioxetine was as high as 99%, and no serious adverse reactions caused by other drugs competing with vortioxetine for plasma proteins have been found, which should be paid attention to together with vortioxetine’s inhibition of CYP450 enzymes *in vivo*. Other antipsychotic drugs inhibited CYP450 enzymes as well. For example, the novel atypical antipsychotic drug asenapine is mainly metabolized in the body by CYP1A2, CYP2D6 and CYP3A4. *In vitro*, it was found that asenapine significantly inhibited CYP1A2, CYP2D6, CYP3A4, and the data of CYP1A2 and CYP2D6 were likely to speculate that the same reaction will occur *in vivo* (Wójcikowski et al., 2020). If inhibitors of the corresponding enzymes or metabolites of enzymes are taken together in the body, it is likely to cause adverse reactions by DDI. In another study, the interaction relationship between iloperidone and lurasidone *in vitro* was found, and it is very likely that it can occur *in vivo* (Danek et al., 2020).

In this paper, the studies we did were *in vitro* on the inhibitory effect of vortioxetine on CYP450. Based on the inhibition mechanism discussed above, it is helpful to treat different conditions encountered by different patients, and it also provides an indispensable basis for the research of *in vivo*.

## 5 Conclusion

In summary, we investigated the inhibitory mechanism and Ki values of vortioxetine on CYP3A4/3A, CYP2B6/CYP2B, CYP2C8/CYP2C-1, CYP2C9/CYP2C-2, CYP2C19/CYP2C-3, CYP2D6/CYP2D and found no TDI effect of vortioxetine on CYP450. In HLMs, CYP3A4 and CYP2C8 were noncompetitive inhibition, CYP2B6 and CYP2C9 were mixed inhibition, and CYP2C19 and CYP2D6 were competitive inhibition. In RLMs, CYP2C-3 was noncompetitive inhibition, CYP2C-1 was mixed inhibition, CYP2B and CYP2C-2 were competitive inhibition, and CYP3A and CYP2D were uncompetitive inhibition. All data need to be supported by future *in vivo* experimental data and provide guidance for the safe use of vortioxetine in clinical practice.

## Data Availability

The original contributions presented in the study are included in the article/Supplementary material, further inquiries can be directed to the corresponding authors.
